# Use of Uncertainty Calculation Software as a Didactic Tool to Improve the Knowledge of Chemistry Students in Analytical Method Validation

**DOI:** 10.1021/acs.jchemed.3c00102

**Published:** 2023-12-15

**Authors:** Maria Cerrato-Alvarez, Samuel Frutos-Puerto, Eduardo Pinilla-Gil

**Affiliations:** † Departamento de Química Analítica, Universidad de Extremadura, Av. de Elvas s/n, 06006 Badajoz, Spain

**Keywords:** Analytical Chemistry, Metrology, Uncertainty, Orthogonal Regression, Low-cost Method, Field Validation Tool

## Abstract

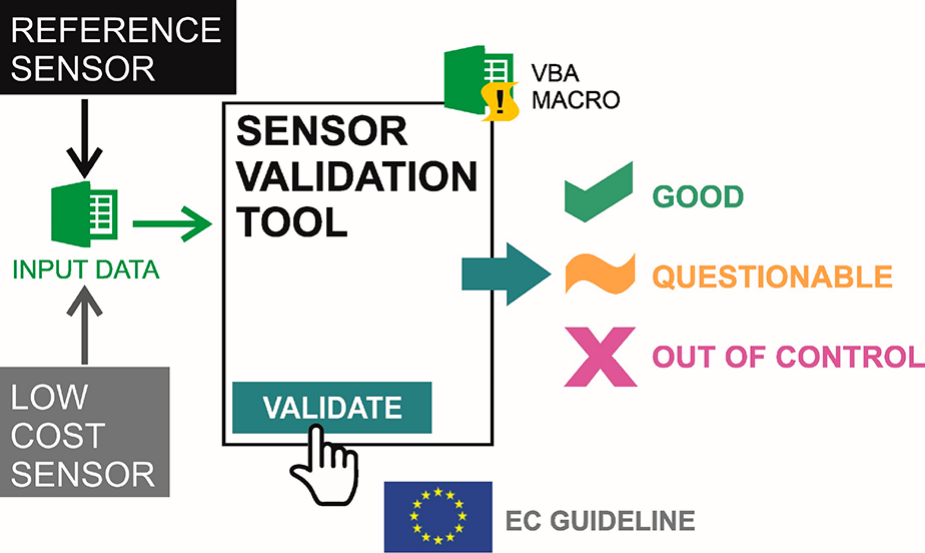

Calculating analytical uncertainties as a part of method validation is a relevant aspect of field and laboratory practices in instrumental analytical chemistry subjects, which usually require complex algorithms. This work describes the development and didactic use of an automatic and straightforward informatics tool, implemented in an Excel macro, for calculating and interpreting the uncertainty of an analytical method against a reference method on field measurements. The software was initially developed for field testing of low-cost air quality monitoring analytical methods against reference methods, and the present work shows its adaptation to a didactic environment. The uncertainty calculation software was implemented through an Excel macro based on Visual Basic as a graphical user interface. It finds a best-fit line that describes the relation between concentrations determined by the candidate and reference methods. The software generates the analytical validation results (slope and intercept with their respective confidence limits, and expanded uncertainty of a concentration determined by the candidate method), hiding the intermediate functions and calculations. The Excel interface eases uncertainty calculations for undergraduate students, although the background mathematics can be quickly unveiled to students for didactic purposes. This tool has been applied to a laboratory exercise focused on validating experimental results obtained in the measurement of ozone levels in ambient air by passive sampling and spectrophotometric detection. The uncertainty calculation software has proved valuable by providing the student a resource to check the analytical quality of the data generated in the laboratory, while assimilating the fundamentals behind the calculations.

## Introduction

One of the main objectives of undergraduate training in Analytical Chemistry is that the student learns to recognize it as the metrological science that develops, optimizes, and applies measurement processes to obtain quality bio(chemical) information from natural and artificial systems.^1−4^ The increasing relevance of learning metrology aspects of Analytical Chemistry is evident just considering factors such as, e.g., (a) the increasing participation of analytical chemists in interlaboratory exercises (with the objectives of achieving comparability and harmonization^5^); (b) the replacement of the term “accuracy” (closeness of the agreement between the result of a measurement and a true value of the measurand) in the literature by the conceptually richer approach of “traceability” (property of a measurement result whereby the result can be related to a reference through a documented unbroken chain of calibrations, each contributing to the measurement uncertainty); (c) the replacement of “precision” (the spread of values obtained with repeated measurements on a given specimen) by “uncertainty” (parameter associated with the result of a measurement, that characterizes the dispersion of the values that could reasonably be attributed to the measurand);^6^ or (d) the need to train skilled professionals to work under requirements of ISO 17025 standard, that states the general requirements for the competence of testing and calibration laboratories.^7^ Therefore, the ability of students to handle the appropriate statistical treatment of analytical data has become a fundamental pillar of their analytical training.^8^

Numerous published protocols include guidelines for validating analytical methods,^9−11^ some based on calculating uncertainty.^12^ The estimation of measurement uncertainty is considered one of the main challenges faced by an analyst in the laboratory, as it requires mastering various statistical tools. Several guides and standards dedicated to evaluating analytical method uncertainty have been published, such as the European Guide for demonstrating the equivalence of a non-regulatory method against a standard reference method on field measurements.^13^

The increasing commercial availability of microsensors is creating a new generation of low-cost air quality analyzers aiming to complement standard methods.^14−16^ Generally, these devices are cheap, lightweight, portable, and easy to operate and maintain by non-technical personnel. Another low-cost strategy for air quality monitoring is passive sampling, which is based on time-integrated sampling. Passive samplers are easy to prepare and deploy, do not need energy, and offer higher spatial resolution thanks to miniaturization and instrumental simplification.^17,18^ These methodologies have been routinely used for decades in industrial environments, where the levels measured are relatively high. However, in recent years, they have been extending their applicability to measurements of immission levels^19^ of atmospheric pollutants in ambient air.^20−26^ In any case, it is essential to validate the performance of these methodologies, as they are typically less reliable than reference methods.^27^ In this context, the scientific and technical community within the air quality monitoring research and commercial sectors agree on the urgent need for intuitive and straightforward validation protocols. These protocols are crucial to avoid the proliferation of unvalidated air quality data,^28^ significantly distorting the correct estimation of air quality levels.

Environmental education plays a critical role in creating a more aware, engaged, and capable society that can address environmental challenges. In the literature, some educational projects on measuring outdoor and indoor air quality using commercial sensors^16,18,29−31^ and passive air samplers^32,33^ have been described. However, exercises based on the interpretation and visualization of atmospheric data are still scarce,^34^ and no didactic tools are available to assist students in the correct validation of these low-cost analyzers.

In this work, we propose a didactic resource for the student’s training in calculating and interpreting uncertainty as a criterion for validating low-cost analytical methods designed to obtain decentralized information about air pollution against a reference method. The proposed statistical tool (Excel macro) has been implemented as a didactic resource in the field and lab practices of the degree in Environmental Science. The students used the macro to validate their results in a field and laboratory exercise for the determination of tropospheric ozone in ambient air using a low-cost method.

## Students Learning Goals

Overall, this laboratory exercise allows students to reinforce their experience in basic concepts of analytical method development and gain experience in the validation and interpretation laboratory results. At the end of the practical, the student should be able to do the following:

(1) Construct calibration curves using solutions of known concentration of indigotrisulfonate.
(2) Calculate ozone concentrations in ambient air from the passive samplers data.
(3) Calculate the expanded uncertainty of a low-cost analytical method against a reference method on field measurements using the validation tool.
(4) Evaluate the analytical performance of a low-cost method using European guidelines for field validation of air pollution measurement methods.

## Theory of Uncertainty Calculation

The uncertainty calculation software for field testing of the low-cost methodology for measuring air pollution levels in ambient air has been developed according to the European Guide for demonstrating the equivalence of a candidate method (non-reference) against a standard reference method.^13^ The equivalence test considers the measurement of the uncertainty as the sum of the uncertainty due to the variability of measurements between two equal candidate samplers/instruments measuring in parallel (if available) plus the uncertainty due to the lack of fit between the candidate method and the reference analyzer measurements, eq 1.
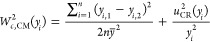
1Where *W*_*c*,CM_^2^ (at the maximum value of the series) is the square of the combined standard relative uncertainty, where *y*_*i*__,1_ and *y*_*i*__,2_ are the results of parallel measurements for a single paired data period *i*, *n* is the number of parallel measurements results, and *y̅* is the average of all the experimental results. *u*_CR_^2^(*y*_*i*_) (at the maximum value of the series) is the square of the uncertainty as a function of concentration (*x*_*i*_) of the candidate method from comparison with the reference method.

The relative expanded uncertainty of the sampler/instrument at 95% confidence was then calculated according to eq 2:
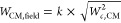
2where the coverage factor is typically *k* = 2. For more information about the statistical details of the uncertainty calculation, please refer to Section 1 of the Supporting Information.

The relative expanded uncertainty *W*_CM__,field_ (%) is compared with the maximum value of relative expanded uncertainty acceptable for using a sampler/instrument for measuring air quality in Europe, published in the European Directive 2008/50/CE (Table 1).^35^ If the *W*_CM__,field_ (%) is lower than the relative uncertainty objectives for data quality set out in Directive 2008/50/CE, the candidate method shall be considered equivalent to the reference method.

**Table 1:** Data Quality Uncertainty Objectives Established by Directive 2008/50/CE^35^

compound	maximum permissible expanded (*k* = 2) relative uncertainty for a fixed measurement (%)
NO_2_	15
O_3_	15
CO	15
PM10	25
PM2.5	25

## Uncertainty Calculation Software

The uncertainty calculation software developed was implemented by an Excel macro, which uses Visual Basic as programming language. It incorporates all the necessary algorithms to apply the European guidelines for field validation of air quality methods,^13^ as described in detail in the Supporting Information.

**Figure 1:**
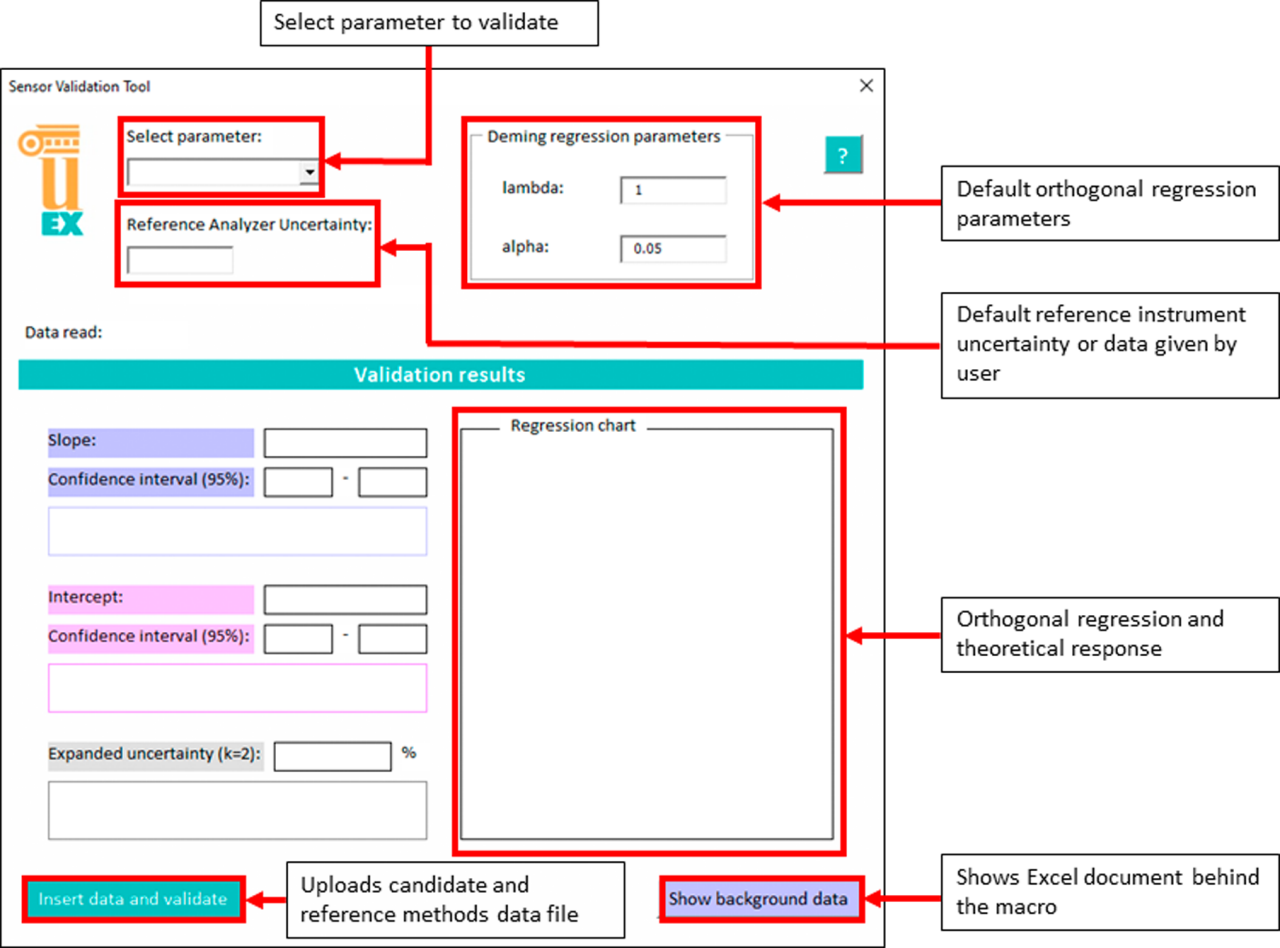
Graphical user interface of the validation tool.

Figure 1 shows the graphical user interface displayed when the macro is started, hiding intermediate functions and calculations from the user, which makes it easier for undergraduate students. Background calculations can be quickly unveiled to students for didactic purposes.

The software allows the selection of the analytical data to be validated (gaseous pollutants as O_3_, CO, and NO_2_; particulate matter as PM10 and PM2.5; and meteorological data as temperature and relative humidity). Temperature and relative humidity serve as guiding parameters because experimental results from low-cost sensors are generally in good agreement with reference data.

The uncertainty of the reference analyzer may be manually inserted, or the user may use a default value. We have assigned the default values based on commercial and technical information for the most common reference analyzers. The tool also allows one to change the Deming regression (also known as orthogonal regression) parameters (lambda and alpha) or use default values. The default parameters alpha (95% confidence interval) and lambda (variance of the reference data series divided by the variance of the candidate method data series) are 0.05 and 1, respectively.

The “Insert data and validate” button allows the user to insert a .xls file with the paired data set to be analyzed (first column for the reference data, second column for candidate sampler/instrument, and third column for a second candidate instrument if available). The tool accepts any set of paired data (10 min average, hourly or another). However, it is advisible to use hourly data in line with official guidelines for low-cost sensor validation. If only one sampler/instrument data column is available, the “*W*_*bs*_^2^” parameter is set to zero. The current version of the uncertainty calculation software is limited to 500 paired data rows. If the source data contains more than 500 rows, the validation tool ignores the data exceeding the limit.

Once the user inserts the data file, the tool performs an orthogonal linear regression of the candidate method concentrations against the corresponding reference method concentrations. It gives the slope and the intercept of the orthogonal regression with their respective confidence limits (95%) and an advice message to the user based on the calculated confidence limits (Table 2).

**Table 2:** Advice for the User Based on Confidence Limits of the Orthogonal Regression

parameter	result	message
slope	interval marked by the confidence limits contains the value 1	slope indicates no systematic error in the candidate-method concentrations (95% confidence level)
slope	interval marked by the confidence limits does not contain the value 1	slope indicates systematic error in the candidate-method concentrations (95% confidence level)
intercept	interval marked by the confidence limits contains the value 0	intercept indicates no systematic error in the candidate-method concentrations (95% confidence level)
intercept	interval marked by the confidence limits does not contain the value 0	intercept indicates systematic error in the candidate-method concentrations (95% confidence level)

The software also generates a graph with the orthogonal regression, the field combined relative expanded (*k* = 2) uncertainty determined by the candidate method at the highest observed concentration, and an advice message informing the user if the sampler/instrument meets the requirements of the European Directive 2008/50/CE on air quality (Table 3).^35^

**Table 3:** Advice for the User Based on Calculated Uncertainty^a^

parameter	uncertainty	uncertainty	uncertainty
temperature	from 0 to 15	from 16 to 30	higher than 30
relative humidity	from 0 to 15	from 16 to 30	higher than 30
O_3_	from 0 to 15	from 16 to 30	higher than 30
NO_2_	from 0 to 15	from 16 to 30	higher than 30
CO	from 0 to 15	from 16 to 30	higher than 30
PM10	from 0 to 25	from 26 to 50	higher than 50
PM2.5	from 0 to 25	from 26 to 50	higher than 50
message	good	questionable	out of control

a The relative expanded uncertainty (%).

**Figure 2:**
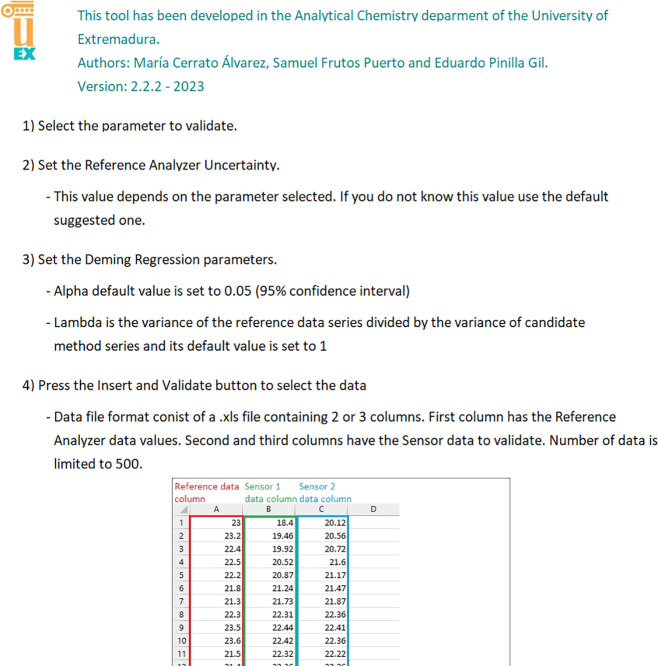
On-screen instructions for using the validation software.

The graphical user interface includes a “Help” button in the upper right corner that sends the users to the guiding screen shown in Figure 2. The screen shows the step-by-step user instructions, including input data format (.xls) to be used as the data source for the validation process.

## Laboratory Exercise: Tropospheric Ozone Determination in Ambient Air by Passive Sampling and UV–Vis Spectrophotometric Detection

The students used the uncertainty validation software to validate their results after field and laboratory practice. They aimed to measure tropospheric ozone levels in ambient air by passive sampling followed by spectrophotometric detection. The practice was carried out by 12 junior students enrolled in the subject “Analytical Techniques for the Pollution Assessment” during the 2021–2022 academic year. The subject belongs to the Environmental Sciences degree at the University of Extremadura. After training on uncertainty and its calculation, the students performed the field (sampling) and laboratory (sample pretreatment and analysis). Then, the students calculated and interpreted the results using the uncertainty validation software. A user experience survey on the software completed the assessment.

The 12 students were divided into 2 groups (6 in each group). Each group was separated into 4 workplaces, where the first two places were occupied by two pairs of students, and the remaining two were occupied by one student each.

## Laboratory Experiment Description

Different methodologies based on various analytical techniques, such as spectrophotometry, fluorescence, and chemiluminescence, have been developed for measuring tropospheric ozone levels in ambient air.^24^ The standard instruments provide reliable continuous data appropriate for air quality regulatory purposes; however, the instrumentation is relatively costly, oversized, and heavy, also requiring significant maintenance costs.^36^ The most used reference methodology in air quality surveillance networks is based on monitoring ozone absorbance in the UV region.^37^ There are low-cost alternative methods based on passive sampling, where ozone is captured by diffusion to a membrane impregnated with a specific chemical reagent that reacts with ozone. The product formed or the remaining reagent is analyzed by a suitable analytical technique.^24,25,38^

**Figure 3:**
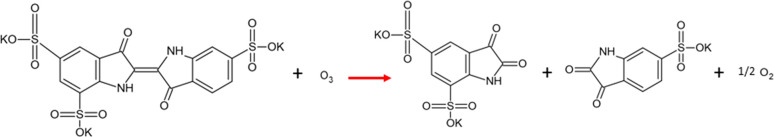
Reaction of ITS with ozone.

In this laboratory exercise, the students applied a passive sampling method based on ozone reaction with the blue reagent indigotrisulfonate (ITS). The reaction generates a nearly colorless product according to a 1:1 stoichiometry (Figure 3). The moles of ITS consumed (measured by the decrease in absorbance of the reagent at 600 nm) equals the moles of ozone sampled, which are related to its concentration in ambient air by Fick’s law of diffusion, eq 3.^38^
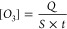
3where [O_3_] is the ozone concentration (μg m^–3^) in ambient air, *Q* is the ozone mass reacted with ITS during the sampling time (as calculated stoichiometrically from the amount of ITS consumed), *S* is the sampling rate provided by the passive sampler manufacturer (*S* = 21.8 × 10^–6^ m^3^ min^–1^) and *t* is the sampling time (min).

The students prepared a 1000 mg L^–1^ ITS stock solution in 50% ethylene glycol: distilled water, by dissolving 0.025 g of ITS in a flask (25 mL) containing 12.5 mL of ethylene glycol and 12.5 mL distilled water. From this, a 20 mg L^–1^ ITS working solution was prepared by diluting 1 mL of stock solution with 49 mL of distilled water. Then, six standards were prepared from the working solution (1, 2, 4, 8, 16, and 20 mg L^–1^) for calibration curve (linear regression using least-squares method), and the absorbances of the standards were measured at 600 nm.

**Figure 4:**
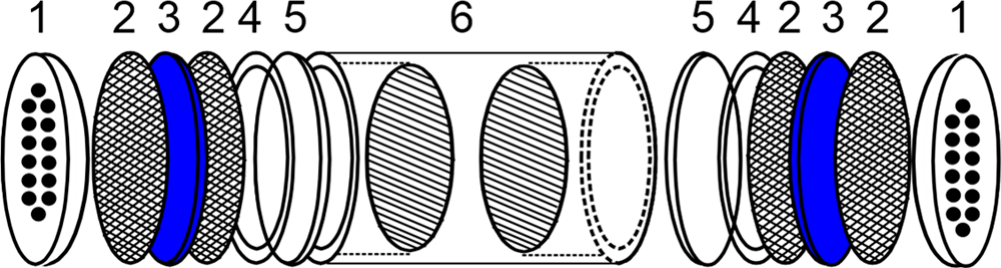
Owaga passive sampler. (1) diffuser end-cap, (2) stainless steel meshes, (3) collection pad, (4) Teflon ring, (5) Teflon disc, and (6) sampler body with two independent chambers.

Commercial Owaga passive samplers (Owaga, USA) were used for sampling. Each workplace has two passive samplers, one for the real sample and one for the blank. The students washed with distilled water and dried all components of the passive sampler, whose design is shown in Figure 4. Thirty microliters of the 1000 mg L^–1^ ITS stock solution was deposited on each collection pad, and the sampler was assembled.

One of the two samplers was exposed to ambient air for approximately 24 h (noting the exact start and end time of sampling). The other sampler was kept in a sealed opaque box for the same time (blank). Sampling was performed next to the air quality monitoring unit (belonging to the Government of Extremadura) located at the University of Extremadura Badajoz campus. The unit is equipped with a ThermoFisher 49i reference photometry analyzer. Thus, paired ozone concentrations data are available for both methods, allowing us to calculate the uncertainty of the method by applying the uncertainty validation software.

After sampling, the students collected the samplers and transferred them to the lab. Then, they disassembled the samplers by removing the two collection pads and depositing them in 10 mL flasks containing approximately 7 mL of distilled water. The flasks were sonicated, made up to volume, and the absorbance was measured with a Jenway 7315 spectrophotometer (1 cm path length). The same procedure was repeated with the samplers used as blanks.

The ozone mass reacted with ITS (*Q*) was calculated from the difference between ITS masses obtained by the blank sampler and the exposed sampler (obtained by external calibration with known concentrations of ITS). Finally, the ozone concentration in ambient air was calculated using eq 3. The experimental procedure provided to the students is shown in Section 2 of the Supporting Information. The analytical quality of the methodology used by the students has been proven by research work done by Garcia et al.^38^ and by our research group.^24,25^

## Hazards

Potassium indigotrisulfonate (CAS no. 67627-18-3) is not considered hazardous. However, in case of skin contact, wash with soap and plenty of water. Ethylene glycol (CAS-No. 107-21-1) is harmful if swallowed and may cause damage to organs (kidney) after prolonged or repeated exposure. In addition, it irritates the skin and should be washed thoroughly after contact. Waste must be disposed of following environmental regulations. Personal protection: laboratory coat, nitrile or latex gloves, and safety glasses.

## Student Work

**Figure 5:**
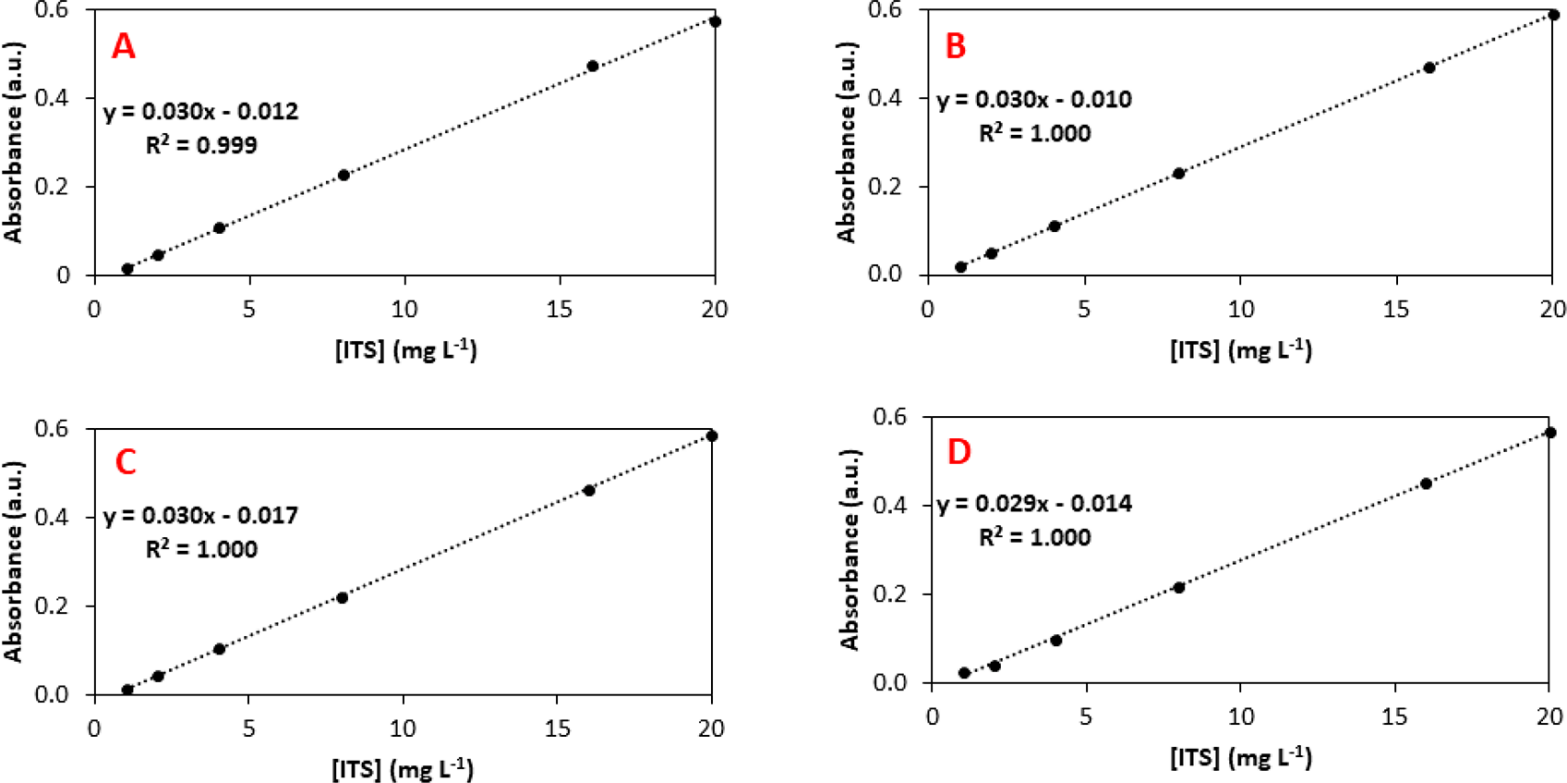
ITS calibration curves in the concentrations range from 1 to 20 mg L^–1^ for (A) workplace 1, (B) workplace 2, (C) workplace 3, and (D) workplace 4.

The student’s work began in the analytical chemistry laboratory. They worked using a written protocol describing the practice, detailed in Section 2 of the Supporting Information. The first step was washing all the laboratory materials and preparing the required solutions (stock and working solutions). Next, each student pair performed the curve calibration based on the absorbances of the six ITS standards. Figure 5 is an example of ITS calibration curves generated by students group 1. The dots represent absorbance data measured by the spectrophotometer, and the dashed line indicates the linear fit using the least-squares method. All data sets show good linearity (*R*^2^ values ≥ 0.999) over the concentration range studied, fulfilling the Beer–Lambert Law. In addition, the slope of each calibration curve is similar (slopes = 0.03).

Then, the students loaded the collection pads, assembled the passive samplers, and exposed one of the two passive samplers to ambient air. This task was carried out in the laboratory for approximately 4 h. After sampling (1100 min later; date and time of start and end of sampling student group 1: 27/04/2022, 06:40 pm to 28/04/2022, 01:00 pm; date and time of start and end of sampling student group 2: 28/04/2022, 06:20 pm to 29/04/2022, 12:40 pm), the pupils extracted the ITS from the collection pads and measured the absorbance. Table 4 shows the average ozone concentrations obtained by the students and reference analyzer (averaged to the same time frame as that used for passive sampling). The results illustrate that the values found by students group 1 (workplaces 3 and 4) and students group 2 (workplace 2) are significantly lower than those measured by the reference analyzer. In contrast, the other values obtained are similar.

**Table 4:** Comparison of Ozone Concentrations Obtained by Students and Reference Analyzer

students group 1	O_3_ (μg m^–3^) passive sampling	O_3_ (μg m^–3^) reference analyzer
workplace 1	56.33	63.40
workplace 2	53.36	
workplace 3	37.99	
workplace 4	37.05	

students group 2	O_3_ (μg m^–3^) passive sampling	O_3_ (μg m^–3^) reference analyzer
workplace 1	60.05	67.10
workplace 2	25.50	
workplace 3	67.55	
workplace 4	53.44	

After the experimental work, the second stage of the practice consisted of interpreting the results and preparing the laboratory report containing the experimental results and validation outcome from the uncertainty calculation software. The instructor provided the students with the ozone data measured by the reference analyzer, and they shared the measured ozone concentrations. In addition, the instructor provided ozone concentration pairs measured in previous years’ courses to have a more significant number of paired data on ozone concentrations (the current database contains 27 data pairs).

**Figure 6:**
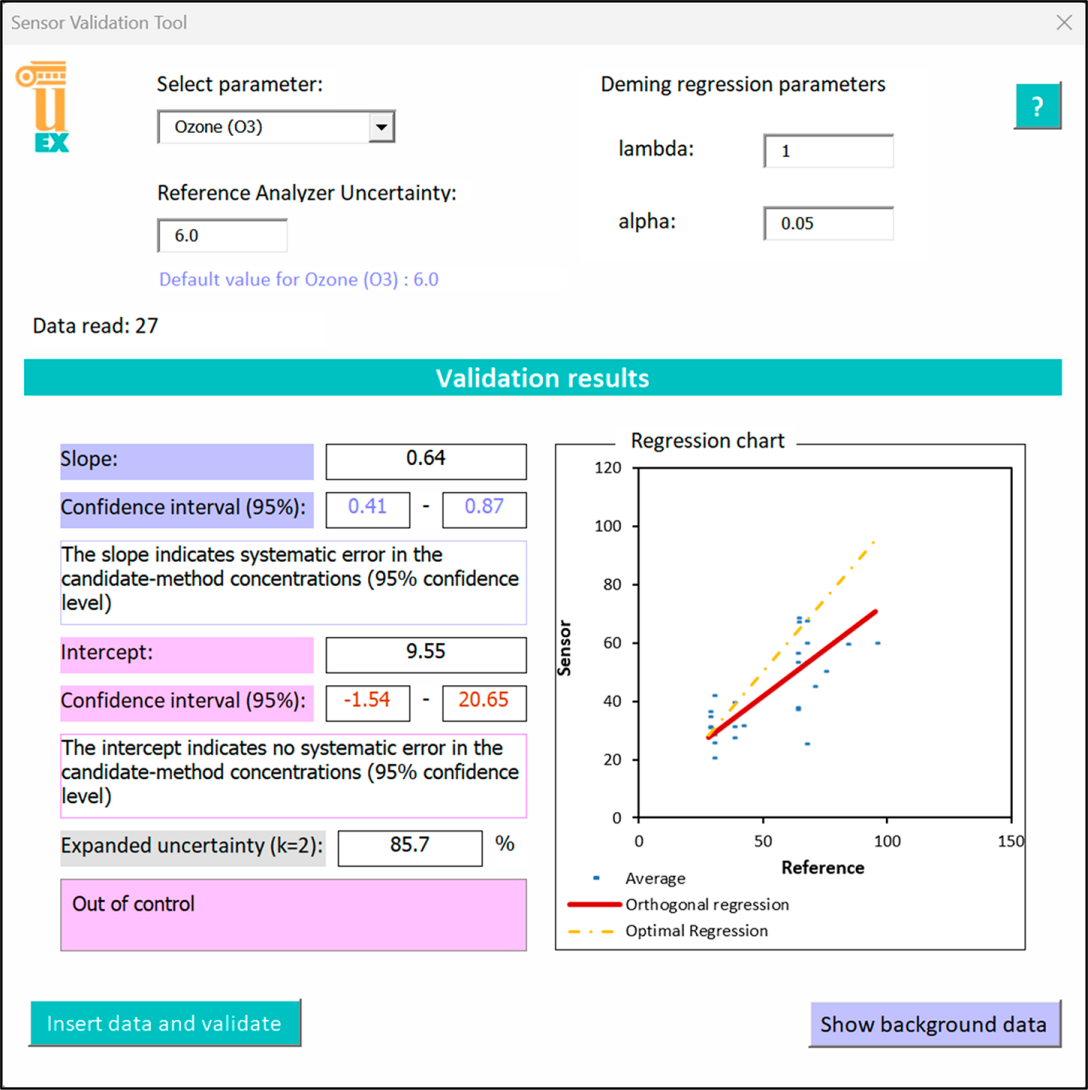
Results of method validation.

The students accessed the uncertainty validation software and inserted the .xls file with the ozone data measured by the reference analyzer (first column) and the ozone data measured during the practice (second column). Once the data file was inserted, the software executed the statistical protocol and returned the validation results. Figure 6 shows an example of the validation results for a given ozone data set. In this case, the somewhat low value of the slope reveals a systematic error in the candidate method since value one is not included within the 95% confidence limit interval. This behavior may be related to excessive reagent depletion when the experiments are carried out during high-level tropospheric ozone sampling periods, a fact that we highlight to the students as an illustration of the importance of the concept of linear range limit in an analytical method. In accordance with the intercept, no systematic error is detected in the candidate method since the value 0 is included within the 95% confidence limits interval. The relative expanded uncertainty to the highest measured value is 85.7%, significantly higher than the maximum value allowed by EU Directive 2008/50/CE,^35^ which is 15% for fixed and 30% for indicative measurements. The orthogonal regression graphic shows the orthogonal regression line (red line) and the ideal result that is expected if there is no systematic error in the candidate method (dashed yellow line), allowing the students to estimate at first glance the overall performance of the method they applied. For more information, please refer to Section 1 of the Supporting Information.

## Discussion

Although the uncertainty calculation software was primarily developed in the context of a research project (Interreg Sudoe NanoSen-AQM^39^), we found it helpful as a didactic resource to validate the results generated in the laboratory exercise described above. So far, most of the lab exercises described in the literature have been validated by hypothesis tests, such as the paired *t* test, in which the result obtained is qualitative;^40^ however, the validation tool developed in this work offers a quantitative result.

After the practice, the supervisors analyzed the student reports, finding clear evidence that they had achieved the learning outcomes. Specifically, 100% of the students were able to construct a calibration curve (linear regression by least-squares method) using Microsoft Excel as software. All students obtained a good fit with a determination coefficient above 0.99. The majority of students (91.7%) correctly calculated ozone concentrations in ambient air. Moreover, the students compared the ozone concentrations obtained with passive sampling with the ozone values measured by the reference analyzer to calculate the accuracy of the measurement as relative error. This outcome was achieved by 75% of the students. The students also demonstrated (83.3%) that they understood the uncertainty calculation for the evaluation of a low-cost method versus on a reference method.

Finally, the students were given a survey to evaluate their perception as users of the uncertainty calculation software. The results of the survey are presented in Table 5. In general, the students consider the software an easy and valuable tool that facilitates the interpretation of the quality of the results obtained in laboratory practice. However, some students complained that the operating instructions provided by the help section should be improved. Also, they considered that the validation parameters provided by the results (slope and intercept of the orthogonal regression, with their corresponding confidence limits and the estimated uncertainty value) should be better explained. Considering this survey and the positive results obtained from this pilot experience, we intend to improve some aspects of the tool to continue applying it in other laboratory exercises in future courses.

**Table 5:** Results of the Survey on the Use of the Validation tool^a^

students’ responses (%)	questions with optional answers
	Q1. In your opinion, how useful is the validation tool?
0	I do not see any benefit
8.3	It has been of little benefit to me
91.7	I find it quite useful
	Q2. What is your opinion on the instructions provided by the validation tool?
0	I did not understand the instructions
41.7	I have partially understood the instructions
58.3	I have understood all the instructions
	Q3. In your opinion, how user-friendly is the validation tool?
0	Very difficult
25.0	Difficult
58.3	Easy
16.7	Very easy
	Q4. What do you think about the clarity of the results shown by the validation tool?
16.7	I did not understand what the results mean
66.7	I partially understood what the results mean
16.7	I perfectly understood what the results mean
	Q5. Has the validation tool improved your ability to interpret the result generated in the practical exercise “Determination of ozone in ambient air by passive sampling and spectrophotometry detection”?
25.0	No
75.0	Yes
	Q6. In your opinion, what aspects of the validation tool could be improved? We welcome suggestions for improvement

a *N* = 12.

## Conclusions

Uncertainty calculation software (Excel macro) has been proved to be a valuable didactic resource to facilitate the understanding of analytical uncertainty, a relatively hard-to-understand concept for undergraduates. The software was tested on a group of 12 students with satisfactory results. It was used to validate the measurement results of ambient air ozone concentrations during a practice against a reference method. The proposed didactic resource makes the statistical handling of data easier, providing a quick and simple method to test the analytical quality of the method used. Given the very low cost of some commercially available air quality analyzers and the wide availability of reference data from standard air quality monitoring units (belonging to official air quality surveillance networks), the proposed tool can be easily implemented as a beneficial didactic resource in practical activities for undergraduate subjects related with instrumental analytical chemistry. The readers interested in testing and using the software with their students can download the Excel macro from the Supporting Information. We are working on implementing the software as a smartphone application.

## Supporting Information Available

Uncertainty calculation, handout provided to student with the lab exercise, instructor notes for calculating ozone concentration, and student survey (PDF, DOCX)
Calculations for solutions preparation and data for ozone concentration calculations in ambient air (XLSX)
Excel Macro for data validation (ZIP)
