# Supplementary material for: Use of Uncertainty Calculation Software as a Didactic Tool to Improve the Knowledge of Chemistry Students in Analytical Method Validation
Source: J Chem Educ. 2023 Dec 15;101(1):104–12. doi: 10.1021/acs.jchemed.3c00102 (PMC10865931; doi:10.1021/acs.jchemed.3c00102)
Supplement: Supplementary file 2 — ed3c00102_si_002.docx [file ed3c00102_si_002.docx]

SupPorting information

Use of uncertainty calculation software as a didactic tool to improve the knowledge of Chemistry students in analytical method validation

Maria Cerrato-Alvarez,^1,*^ Samuel Frutos-Puerto,^1^ Eduardo Pinilla-Gil ^1^

^1^ Departamento de Química Analítica, Universidad de Extremadura, Av. de Elvas s/n, 06006, Badajoz, Spain

*Corresponding author e-mail: macerratoa@unex.es

This document includes:

Uncertainty calculation……………………………………………………………………………….2

Handout provided to student with the lab exercise………….…………………………………4

Instructor notes for calculating ozone concentration………………………………………….8

Student survey………………………………………………………………………………………..10

**SECTION 1. UNCERTAINTY CALCULATION**

The European Guide for the demonstration of equivalence of ambient air monitoring methods considers uncertainty as the sum of the uncertainty due to the variability of measurements between two equal candidate samplers/instruments measuring in parallel (if available) plus the uncertainty due to the lack of fit between the candidate method and the reference analyzer measurements.

*Between-samplers/instruments uncertainty*

The square of the relative standard uncertainty due to the variability of measurements between samplers/instruments ($W_{bs}^{2}$) is calculated from the difference between the measurements of the samplers/instruments measuring in parallel, according to Eq. (S1):

$W_{bs}^{2}=\frac{\sum_{i=1}^{n} \left( y_{i,1}-y_{i,2} \right)^{2}}{2ny^{̅2}}$ (S1)

where $y_{i,1}$ and $y_{i,2}$ are the results of parallel measurements for a single paired data period i, $n$ is the number of parallel measurements results and $y^{̅}$ is the average of all the experimental results.

The relative standard uncertainty between samplers/instruments is the square root of $W_{bs}^{2}$. The relative standard uncertainty as a per cent is obtained by multiplying by 100. According to the European Guide mentioned above, the relative standard uncertainty (%) should not exceed 5%.

*Comparison with the reference method*

Regarding the uncertainty due to the lack of fit between the candidate method and the reference analyzer, it is assumed that the relationship between the measurements obtained by the candidate method (y) and the measurements obtained by the reference analyzer (x) can be described by a linear relation of the form Eq. (S2).

$y_{i}=a+{bx}_{i}$ (S2)

The linear correlation was evaluated using the orthogonal regression technique (also known as Deming regression), this being preferable to standard regression since it includes the uncertainty of x-axis values. The square of the sampler/instrument uncertainty ($u_{CR}^{2}\left( y_{i} \right)$) as a function of concentration ($x_{i}$) was calculated from Eq. (S3):

$u_{CR}^{2}\left( y_{i} \right)=\frac{RSS}{\left( n-2 \right)}-u^{2}\left( x_{i} \right)+\left[ a+\left( b-1 \right)x_{i} \right]^{2}$ (S3)

where $a$ and *b* are, respectively, the intercept and the slope of the orthogonal regression, $n$ is the number of parallel measurements results, $u\left( x_{i} \right)$ is the standard uncertainty of the reference analyzer (obtained from the analyzer specifications or experimental measurements with two reference analyzers in parallel), and $RSS$ is the sum of the absolute (Eq. S4) or relative (Eq. S5) residuals resulting from the orthogonal regression.

$RSS=\sum_{i=1}^{n} \left( y_{i}-a-bx_{i} \right)^{2}$ when $\left( y_{i}-a-bx_{i} \right)^{2}$ is constant (S4)

$RSS=\left( a+{bx}_{i} \right)^{2}\sum_{i=1}^{n} \left( \frac{y_{i}}{a+bx_{i}}-1 \right)^{2}$ when $\left( \frac{y_{i}}{a+bx_{i}}-1 \right)^{2}$ is constant (S5)

*Calculation of the combined uncertainty of candidate method*

The square of the field combined standard relative uncertainty $W_{c,CM}^{2}$  (at the maximum value of the series) is calculated by adding $W_{bs}^{2}$ plus $u_{CR}^{2}\left( y_{i} \right)$ (at the maximum value of the series), using Eq. (S6):

$W_{c,CM}^{2}(y_{i})=\frac{\sum_{i=1}^{n} \left( y_{i,1}-y_{i,2} \right)^{2}}{2ny^{̅2}}+\frac{u_{CR}^{2}\left( y_{i} \right)}{y_{i}^{2}}$ (S6)

*Calculation of the expanded uncertainty of candidate method*

The relative expanded uncertainty of the sampler/instrument at 95% confidence was then calculated according to Eq. (S7):

$W_{CM, field}=k\cdot\sqrt{W_{c, CM}^{2}}$ (S7)

where the coverage factor is typically $k$= 2.

**SECTION 2. HANDOUT PROVIDED TO STUDENT WITH THE LAB EXERCISE**

DETERMINATION OF OZONE IN AMBIENT AIR BY PASSIVE SAMPLING AND DETECTION BY MOLECULAR ABSORPTION SPECTROPHOTOMETRY (VISIBLE)

OBJETIVE

Training in the technique of passive sampling and measurement by UV-Vis molecular absorption spectrophotometry to determine the levels of atmospheric pollutants in ambient air.

1.INTRODUCTION

Tropospheric ozone is a secondary pollutant formed by photochemical reactions of volatile organic compounds (VOCs) and nitrogen oxides (NOx) activated by solar radiation and temperature. In southern Europe and other climatic regions with similar characteristics, tropospheric ozone is a major environmental problem. Continuous exposure to high concentrations of ozone in ambient air can cause damage to public health and vegetation. Current European environmental legislation sets different maximum levels for ambient concentrations of tropospheric ozone for the protection of human health and ecosystems.

Different analytical methodologies have been developed to determine ozone levels in ambient air. The standard methodology commonly used in air quality monitoring networks is based on measuring ozone absorbance in the ultraviolet region by a continuous analyzer provided with a suction pump for air sampling (standard UNE-EN 14625/2013). It is a reliable method, although the equipment required is expensive to purchase and maintain. However, there is a more affordable alternative based on passive sampling in which ozone migrate by diffusion to a membrane impregnated with indigo trisulfonate (ITS), where the reaction shown in Figure S1 occurs.


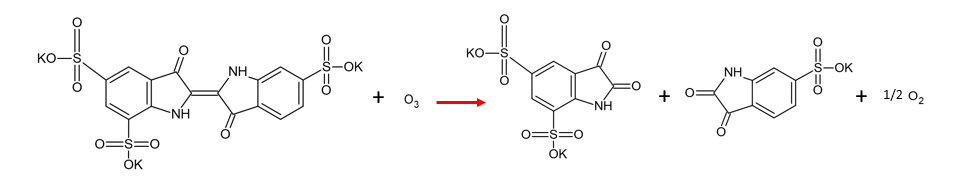


Figure S1. Reaction of ITS with ozone.

The mole amount of ozone that has reacted in a period equals the mole amount of ITS consumed. The latter is measured by the decrease of the reagent’s absorbance at 600 nm.

Once the amount of ozone has been calculated, it is related to its concentration in ambient air by applying Fick’s law, considering that all the ozone has reached the membrane by diffusion. For this purpose, it is necessary to know the sampling rate of the passive sampler, which takes into account its geometry and the diffusion coefficient of the ozone under the ambient conditions of the measurement. The equation for the calculation of the ambient ozone concentration is:

$\left[ O_{3} \right]=\frac{Q}{S\cdot t}$ (S8)

where $\left[ O_{3} \right]$ is ozone concentration (µg m^-3^); $Q$ is the amount of ozone consumed (µg); S is the sampling rate (m^3^ min^-1^) and$t$ is the sampling time (min).

2.EXPERIMENTAL

2.1.SOLUTIONS

-A 1000 mg L^-1^ ITS stock solution, in distilled water:ethylene glycol (50:50). The appropriated amount of the reagent is added to a 25 mL flask containing 12.5 mL ethylene glycol and made up to the final volume with distilled water. Store away from light.

-A 20 mg L^-1^ ITS working solution. Dilute the stock standard solution 1/50 with water in a 50 mL flask. It is prepared just before use.

2.2.SAMPLING WITH PASSIVE SAMPLERS

Each lab group (typically a pair of students) receives two Owaga passive samplers (Figure S2), one for the real sample and one for the blank.


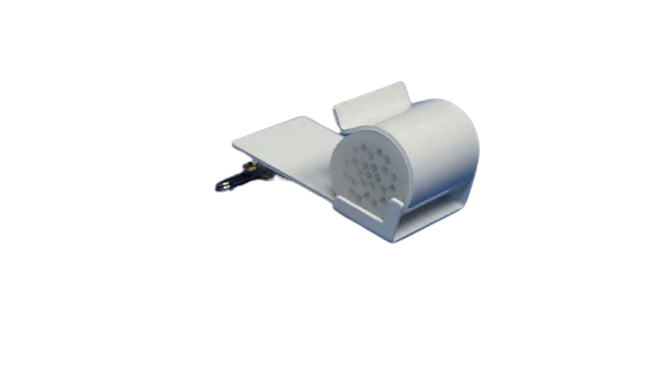


Figure S2. Owaga passive sampler.

Owaga passive samplers are taken and completely disassembled using tweezers, taking care not to mix the parts of both samplers. All the sampler components must be washed with distilled water and dried before each use, except for the collection pads. Figure S3 shows the complete exploded view of an Owaga sampler.


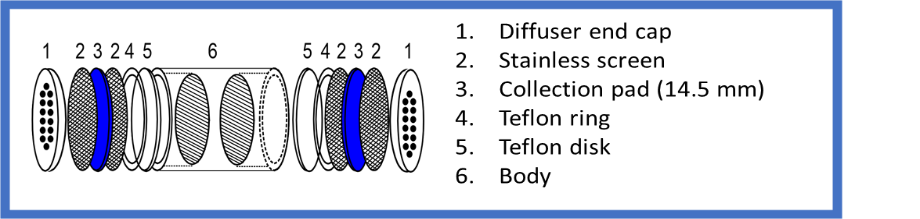


Figure S3. Complete exploded view of an Owaga sampler.

Deposit 30 µL of the ITS stock standard solution (1000 mg L-1) on each of the collection pads, allow to dry and set the sampler using tweezers, according to the procedure shown in Figure S4.


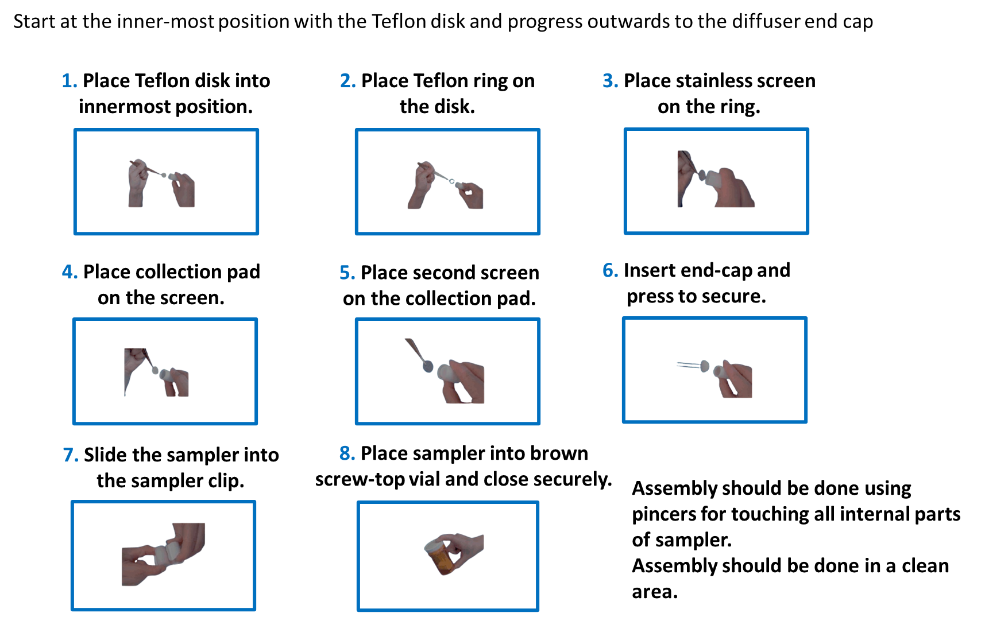


Figure S4. Assembly of the Owaga passive sampler.

Expose one of the two Owaga samplers to ambient air, noting the exact start time of sampling. The other sampler is kept in a sealed opaque box for the same time (blank).

2.3.OZONE DETERMINATION. EXTERNAL STANDARD METHOD

**Procedure for obtaining the calibration curve:**

To five 10 mL volumetric flasks, add increasing volumes of the 20 mg L^-1^ ITS working standard, so that the final volume of the flask contains ITS concentrations between 1 and 20 mg L^-1^, and add water to the mark (Table S1). Record absorbances at 600 nm, using distilled water as a blank.

|  | Table S1. Absorbance data. | | |
| --- | --- | --- | --- |
| mL ITS standard | | mg L^-1^ ITS | A (A.U.) |
|  | | 1.0 |  |
|  | | 2.0 |  |
|  | | 4.0 |  |
|  | | 8.0 |  |
|  | | 16.0 |  |
|  | | 20.0 |  |

**Measurement of real and blank samples:**

Remove the samplers from their places of exposure to the ambient air, noting the exact time of the end of the collection. Dismantle the samplers with tweezers by removing the two pads and placing them together in a 10 mL flask containing about 7 mL of distilled water. Sonicate the flask, make up to the mark with distilled water and measure the absorbance. Repeat the procedure with the sampler used as blank. Record the absorbance As (sample) and Ab (blank).

3.RESULTS

-Graph the calibration curve. Adjust it by the least squares method and check its quality parameters.

-Calculate the concentration of ITS in the real sample (Cs) and the blank (Cb).

-Calculate the ozone mass in micrograms equivalent to the mass of ITS consumed, according to the stoichiometry of the reaction in Figure S1.

-Calculate the ozone concentration in the ambient air according to Eq. S8, considering that the sampling rate for the Ogawa samplers is 21.8 x 10^-6^ m^3^ min^-1^ (manufacturer’s data).

-Calculate the relative standard deviation of the determination, using the set of experimental data obtained in the experimental session.

-Calculate the accuracy of the determination as relative error with respect to the certified value of ozone concentration measured in the air quality monitoring station of Badajoz, belonging to the REPICA network (Red Extremeña de Protección e Investigación de la Calidad del Aire). The instructors will provide this data.

-Calculate the relative expanded uncertainty by the validation tool using the experimental data set obtained in the two experimental sessions and the data measured in previous years’ courses (Table S2).

| Table S2. Experimental data set measured in previous years’ courses. | |
| --- | --- |
| [O_3_]_reference_  (µg m^-3^) | [O_3_]_passive sampler_  (µg m^-3^) |
| 70.36 | 45.10 |
| 83.74 | 59.48 |
| 74.64 | 50.10 |
| 41.68 | 31.63 |
| 95.33 | 59.99 |
| 28.15 | 36.45 |
| 28.15 | 34.72 |
| 28.15 | 31.05 |
| 28.15 | 31.27 |
| 29.82 | 20.69 |
| 29.82 | 41.98 |
| 29.82 | 28.53 |
| 29.82 | 25.69 |
| 37.82 | 31.14 |
| 37.82 | 27.29 |
| 37.82 | 39.52 |
| 64.03 | 67.25 |
| 64.03 | 68.40 |
| 64.03 | 51.03 |

-Compare the experimental ozone concentration value measured with the limit values contemplated in the air quality legislation (Real Decreto 102/2011).

**SECTION 3. INSTRUCTOR NOTES FOR CALCULATING OZONE CONCENTRATION**

It is essential to create organized spreadsheets in Excel to calculate ozone concentrations in ambient air. The instructor can provide students with pre-created spreadsheets for students to complete or can help create them during the lab period. An example of a spreadsheet in .xls format is shown in the Supplementary Information.

Calculations for solutions preparation

Each workplace (composed of two students or one student) prepares the following solutions:

1. ITS stock solution: weigh 25 mg of ITS and add to a 25 mL flask containing 12.5 mL ethylene glycol and made up to the final volume with distilled water.
2. ITS working solution: dilute 1 mL of ITS stock solution with 49 mL of distilled water. Flask volume 50 mL.
3. ITS standard solutions: add increasing volumes of the working solution to five 10 mL flasks (Table S3). Make up to the mark with distilled water.

|  | Table S3. Volumes of standard solutions from the working solution. | |
| --- | --- | --- |
|  | mg L^-1^ ITS | mL ITS standards |
|  | 1.0 | 0.5 |
|  | 2.0 | 1.0 |
|  | 4.0 | 2.0 |
|  | 8.0 | 4.0 |
|  | 16.0 | 8.0 |
|  | 20.0 | -* |

* Use the ready-made working solution.

Calibration curve

1. Record the absorbances of the 6 standards at a wavelength of 600 nm, as shown in Table S1-a of the spreadsheet.
2. Input the absorbance values and the ITS concentrations into a spreadsheet and create a scatter plot (absorbance on the y-axis and concentration on the x-axis). Fit a linear trend line through the data and report the lineal equation (slope and intercept) and determination coefficient (R^2^) using the least squares linear regression method. The R^2^ should be as close to 1 as possible (Table S2-b).

Absorbance real and blank passive sampler

1. Measure the absorbance value for the real sample solution (As) and the blank (Ab), (Table S1-b).

Calculations of ozone concentration in ambient air

The calculations shown below correspond to the Table S3-c.

1. Calculate concentration of ITS extract (mg L^-1^) in the blank using Eq. S9.

$C_{b}=\frac{A_{b}-intercept}{slope}$ (S9)

1. Calculate concentration of ITS extract (mg L^-1^) in the real sample using Eq. S10.

$C_{s}=\frac{A_{s}-intercept}{slope}$ (S10)

1. Calculate mass of ITS extract (µg) in the blank using Eq. S11.

${{Mass}_{b}=C}_{b}\left( \frac{mg}{L} \right)*0.01\left( L \right)*1000(\mu g)$ (S11)

1. Calculate mass of ITS extract (µg) in the real sample using Eq. S12. Flask volume 10 mL.

${{Mass}_{s}=C}_{s}\left( \frac{mg}{L} \right)*0.01\left( L \right)*1000(\mu g)$ (S12)

1. Calculate ITS mass consumed (µg) using Eq. S13.

${ITS mass consumed=mass}_{b}-{mass}_{s}$ (S13)

1. Convert the ITS mass consumed to ozone mass (Q) (µg), taking into account the stoichiometry of the reaction between ozone and ITS is 1:1, Eq. (S14). Molecular weight ITS= 616.72 g mol^-1^; molecular weight ozone= 48 g mol^-1^.

$Q=ITS mass consumed*\frac{1 \mu mol ITS}{616.72 \mu g ITS}*\frac{1 \mu mol O_{3}}{1 \mu mol ITS}*\frac{48 \mu g O_{3}}{1 \mu mol O_{3}}$ (S14)

1. Calculate the ozone concentration in ambient air (µg m^-3^) using Eq. (S8). S is the sampling rate provided by the Ogawa sampler manufacturer (*S* = 21.8 x 10-6 m^3^ min^-1^) and t is the sampling time (min).

**SECTION 4. SURVEY**

Thank you for reporting your user experience of the Macro Sensor Validation Tool (SVT). The results will be treated anonymously. This tool has been developed with funding from the European project NanoSenAQM (Interreg-Sudoe programme) and is currently used in the regional project Comunicaire (funded by the Junta de Extremadura) and in other projects of the AQUIMA research group of the UEx. These projects are co-financed by the European Regional Development Fund (ERDF).

**Q1. In your opinion, how useful is the validation tool?**

- I do not see any benefit
- It has been of little benefit to me
- I find it quite useful

**Q2. What is your opinion on the instructions provided by the validation tool?**

- I did not understand the instructions
- I have partially understood the instructions
- I have understood all the instructions

**Q3. In your opinion, how user-friendly is the validation tool?**

- Very difficult
- Difficult
- Easy
- Very easy

**Q4. What do you think about the clarity of the results shown by the validation tool?**

- I did not understand what the results mean
- I partially understood what the results mean
- I perfectly understood what the results mean

**Q5. Has the validation tool improved your ability to interpret the result generated in the practical exercise "Determination of ozone in ambient air by passive sampling and spectrophotometry detection"?**

- No
- Yes

**Q6. In your opinion, what aspects of the validation tool could be improved? We welcome suggestions for improvement**
